# Buffalo Medical and Surgical Association

**Published:** 1883-10

**Authors:** Clayton M. Daniels

**Affiliations:** Secretary


### Society Reports.

### Buffalo Medical and Surgical Association.

*Regular Meeting, September 4th,* 1883.

President, Dr. J. Cronyn, in the Chair. Dr. C. M. Daniels, Secretary.

Present—Drs. Mann, Hopkins, Peterson, Howe, Frank, Weil, MacNeil, Hayd, Bartlett, Hubbell, Abbott, Brecht, Hartwig, Tremaine, Fowler, Heath, Cary, Fell, and, by invitation, Drs. Park and Southwick.

Upon motion, Dr. Park was invited to participate in the meeting.

Dr. Howe called the attention of the society to the treatment of granular lids with infusions of abrus precatorius, or, as it is commonly called, jequiriti. He referred to a former method sometimes adopted, of inoculating these cases with gonorrhœal pus, which occasionally improved the condition of the eye, but in many instances also destroyed it. The jequiriti produced an inflammation similar to that caused by the pus, but fortunately, its degree could be controlled at will; a decided advantage was thus gained, and for certain cases, this new remedy seems to be full of promise. It is probably not adapted to all varieties of granular conjunctivitis, but principally to those in which the secondary corneal complication (pannus) is in a great degree as compared with the conjunctivitis itself. In illustration of these remarks, a patient was presented, a young man whose left eye was entirely blind from a thick pannus, in spite of the fact that sulphate of copper, nitrate of silver and other ordinary applications had been faithfully used a long while. Such cases can still hope for at least some relief by the jequiriti treatment.

Essay by Dr. H. E. Hayd; subject, "Club-foot." (The paper will appear in the next number of the JOURNAL.)

*Discussion*—Dr. Park stated that he had been especially interested, and thought the points well taken.

Dr. Bartlett thought that Dr. Hayd justly complained of the great expense of the various mechanical appliances used, and thought they might be simplified and cheaper. Had himself used tin shoes and adjustments where patients were poor.

Dr. Frank asked up to what time an operation was advisable.

Dr. Hartwig thought the essayist had clearly explained the conditions, although some joint relations had not been observed; yet, as a whole, the paper presents a clear and forcible argument.

Dr. Cronyn, in referring to the primary cause and theory advanced by Dr. Hayd, said it was a subject that had occupied much attention from many men. He considered the hypothesis of deformity as yet very uncertain, and that there was room for any amount of investigation upon the subject.

Dr. Hayd said he tried to make the paper as practical as possible in order to excite discussion. In reply to Dr. Frank's question, he thought age should not interfere, but to operate as soon as possible. As to the theories advanced, they were entirely his own.

Discussion upon the action of "jequiriti," as explained by Dr. Howe, followed.

Dr. Tremaine asked if *similia similibus curantur* was not proven by its action?

Dr. Bartlett had heard of the intentional inoculation of gonorrhœal virus in certain forms of ophthalmia, and asked if such had been done, and with what result.

Dr. Abbott stated that it had been done, but that results did not prove its value, and that it was not an advisable experiment.

Dr. Howe said it called to his mind the treatment of "acne" by chrysophanic acid, where the irritation was productive of the best results; had treated several cases successfully with it, and that clinically the fact was very interesting. The strength used was twenty grains to one ounce of vaseline.

Dr. Fell spoke of a case of "acne," in an aggravated form, of seventeen years' duration, which was so treated, with a complete cure.

Dr. Cronyn said it had been used a long time, but was considered a dangerous remedy, and he should be extremely cautious about using it.

*Prevailing Diseases* — Dysentery, typhoid fever, follicular pharyngitis and some cholera infantum.

CLAYTON M. DANIELS, *Secretary.*

